# Cost-Effectiveness of Elranatamab Versus Teclistamab for the Management of Patients with Triple-Class Exposed Relapsed/Refractory Multiple Myeloma in Italy

**DOI:** 10.3390/cancers18071070

**Published:** 2026-03-25

**Authors:** Cirino Botta, Giorgio Lorenzo Colombo, Sergio Di Matteo, Chiara Martinotti, Emma Lucia Fogliati, Giacomo Matteo Bruno, Giuseppe Novelli, Roberto Di Virgilio, Barbara Veggia, Sara Galimberti

**Affiliations:** 1 Department of Health Promotion, Mother and Child Care, Internal Medicine and Medical Specialties, University of Palermo, 90100 Palermo, Italy; 2 Department of Drug Sciences, University of Pavia, 27100 Pavia, Italy; giacomomatteo.bruno@unipv.it; 3 CEFAT—Center of Pharmaceuticals Economics and Medical Technologies Evaluation, Department of Drug Sciences, University of Pavia, 27100 Pavia, Italy; 4 Center of Research, SAVE Studi—Health Economics and Outcomes Research, 20100 Milan, Italychiara.martinotti@savestudi.it (C.M.); emma.fogliati@savestudi.it (E.L.F.); 5 Pfizer s.r.l., 00100 Rome, Italy; giuseppe.novelli@pfizer.com (G.N.);; 6 Department of Clinical and Experimental Medicine, Section of Hematology, University of Pisa, 56100 Pisa, Italy; sara.galimberti@unipi.it

**Keywords:** relapsed and refractory multiple myeloma, triple-class exposed, cost-effectiveness, economic evaluation, elranatamab

## Abstract

Background: Multiple myeloma remains an incurable malignancy in which patients ultimately experience relapses and refractory disease despite therapeutic advances. Triple-class exposed relapsed/refractory MM (TCE/RRMM) patients represent both a population with high unmet clinical needs and a substantial economic burden for the Italian National Health Service (NHS). Recently, BCMA-CD3 bispecific antibodies, including elranatamab and teclistamab, have expanded the treatment options for these patients. This study aimed to evaluate the cost-effectiveness of elranatamab versus teclistamab in adults with TCE/RRMM from the Italian NHS perspective. Methods: A partitioned survival model with three health states—progression-free survival, post-progression survival, and death—was developed over a 25-year lifetime horizon, adopting weekly cycles and a 3% annual discount rate. Clinical data for elranatamab were derived from the MagnetisMM-3 trial, while teclistamab outcomes were estimated through matching-adjusted indirect comparisons using MajesTEC-1 data. Direct medical costs included those associated with drug acquisition and administration, disease management, adverse event management, and end-of-life care. Utility values were obtained from EQ-5D-based assessments in MagnetisMM-3. Deterministic and probabilistic sensitivity analyses were performed to test model robustness. Results: Elranatamab yielded 3.31 life-years and 2.33 QALYs per patient, compared with 1.83 life-years and 1.27 QALYs for teclistamab. Total lifetime costs were lower for elranatamab (EUR 153,337) versus teclistamab (EUR 224,610), generating EUR 71,273 in savings. Thus, elranatamab is considered to be dominant, due to its higher efficacy and lower cost. Furthermore, the robustness of these findings was confirmed through sensitivity analyses. Conclusions: From the Italian NHS perspective, elranatamab represents a clinically superior and economically favorable option for patients with TCE/RRMM. The obtained results support its value and sustainability within the national treatment landscape.

## Simple Summary

Using a partitioned survival model from the Italian National Health Service perspective, elranatamab was evaluated against teclistamab in adults with triple-class exposed relapsed/refractory multiple myeloma, incorporating indirect comparative clinical evidence, lifetime costs, and outcomes. Elranatamab was found to be dominant, providing higher life-years and QALYs at lower total costs, with the results remaining robust across sensitivity analyses.

## 1. Introduction

Multiple myeloma (MM) is a hematologic malignancy characterized by clonal plasma cell proliferation in the bone marrow [1,2], representing a relevant clinical and epidemiological burden in Italy. MM accounts for approximately 2% of all cancers and 10% of hematologic malignancies [3], with real-world Italian data reporting an incidence of 9 new cases per 100,000 inhabitants and a prevalence of 40.9 cases per 100,000 [4]. MM remains incurable [5], with an estimated mortality rate of 5.5 cases per 100,000 inhabitants [6].

Therapeutic advances over the past decade, including proteasome inhibitors (PIs), immunomodulatory drugs (IMiDs), and monoclonal antibodies (mAbs) targeting CD38, have improved survival and quality of life (QoL) [7]. Despite these advances, relapse and refractory disease remain inevitable for most patients, resulting in progressively shorter and less durable responses with subsequent treatment lines [8]. The relapsed/refractory multiple myeloma (RRMM) patient population poses substantial clinical and economic challenges [9]. Among these patients, those with triple-class exposed relapsed/refractory multiple myeloma (TCE/RRMM) have received at least three prior lines of therapy, including a PI, an IMiD, and an anti-CD38 monoclonal antibody [4]. In Italy, the prevalence of TCE/RRMM has been estimated at 4.1 cases per 100,000 inhabitants [4], and no clearly defined standard of care currently exists for this population [5].

Novel therapies such as CAR T-cell treatments and BCMA-directed bispecific antibodies have recently expanded therapeutic options for TCE/RRMM [10]. Elranatamab and teclistamab are BCMA-CD3 bispecific antibodies that redirect T cells against malignant plasma cells [11,12]. Their clinical efficacy and manageable safety profiles have been demonstrated in the phase 2 MagnetisMM-3 and MajesTEC-1 trials [11,12], respectively, leading to their regulatory approval by the FDA and EMA and subsequent availability in Italy [10].

MM management is associated with a substantial economic burden for the Italian National Health Service (NHS). A recent Italian real-world study estimated an average annual cost of EUR 119,899 per patient, largely driven by MM-specific drug costs (86%), followed by hospitalizations and outpatient services [4]. These results reflect the complexity of MM management, characterized by frequent relapses, multiple therapeutic lines, and the need for combination regimens across different drug classes, all of which significantly impact the Italian NHS [4]. In this context, the objective of the present analysis was to provide national healthcare authorities with an evaluation of the efficiency of elranatamab compared with teclistamab for the treatment of patients with TCE/RRMM, considering the criteria of the NHS.

## 2. Materials and Methods

A cost-effectiveness analysis was carried out, adopting a partitioned survival model (PSM) developed in Microsoft^®^ Excel to capture disease progression, health states, and events influencing costs and outcomes over time. In particular, this model was used to evaluate the cost-effectiveness of elranatamab versus teclistamab in TCE/RRMM from the Italian NHS perspective.

A lifetime horizon of 25 years was adopted, consistent with the mean age of the population in reference clinical studies [11]. In line with national guidelines, costs and health outcomes were discounted at an annual rate of 3% in the base case analysis [13,14].

The model structure included three health states: progression-free survival (PFS), progression state (PPS, post-progression survival), and death.

The target population reflected that of the phase 2, multicenter, open-label MagnetisMM-3 trial [11], with a median age of 68 years (19.5% aged ≥75 years) and 63.4% with an ECOG performance status of 1–2. Patients had received a median of five prior treatment lines, with 96.7% classified as triple-class refractory and 42.3% as penta-drug refractory. Background mortality was incorporated using data from the Italian National Institute of Statistics [15].

The main effectiveness parameters analyzed were PFS and overall survival (OS). Within the PSM framework, these endpoints were used to directly estimate the proportion of patients in each health state over time. Time to treatment discontinuation (TTD) was also included to account for the time on treatment within the PFS state, distinguishing between progression-free patients remaining on or off therapy.

An OS curve was used to estimate the proportion of the cohort that remained alive over time, which was extrapolated beyond the currently available data to meet the requirement of modeling over the selected time horizon. The area under the extrapolated OS curve provided an estimate of mean life expectancy. In addition, for each health state, a specific cost and QoL adjustment weight (utility) were assigned within each period, enabling calculation of the cumulative costs and cumulative Quality-Adjusted Life-Years (QALYs) over the model time horizon.

Only direct medical costs were included: drug costs (including the primary treatment cost, in turn including pre-medication costs for primary treatments where relevant, subsequent treatment costs, and administration costs), disease management costs (healthcare resources such as visits, hospitalizations, monitoring), adverse event management costs, and end-of-life care costs.

The results are expressed as the Incremental Cost-Effectiveness Ratio (ICER), defined as the incremental cost per QALY gained and assessed against a willingness-to-pay threshold of EUR 33,004 per QALY, reflecting the average acceptability threshold recently published for Italy [16]. To assess the uncertainty of variables and assumptions, and to determine the robustness of the results, deterministic and probabilistic sensitivity analyses were conducted.

The model was developed in alignment with best international practices in health economics, following the Consolidated Health Economic Evaluation Reporting Standards (CHEERS) [17]; notably, the methodological approach and cost and effectiveness data were discussed and approved by clinical experts. The model structure is shown in Figure S1 in the Supplementary Materials.

### 2.1. Input Parameters

#### 2.1.1. Clinical Effectiveness and Safety

The effectiveness and safety data for elranatamab were obtained based on the 28.4-month data from MagnetisMM-3 [18].

As the clinical trial data did not cover the full analytic time horizon, extrapolation of survival outcomes was necessary to inform the model. Accordingly, multiple distributions—including exponential, Weibull, Gompertz, log-normal, log-logistic, gamma, and generalized gamma—were fitted to patient-level data from the 28.4-month MagnetisMM-3 study. Model selection was guided by both statistical fit—assessed through the Akaike Information Criterion (AIC) and Bayesian Information Criterion (BIC)—and visual inspection of the fitted curves for OS and PFS associated with elranatamab.

In the base case scenario, the Weibull distribution was adopted for both OS and PFS curves, as it yielded the best statistical fit (i.e., the lowest AIC/BIC), while alternative distributions were explored in scenario analyses. The proportion of patients in each health state at each time point was determined based on the efficacy of each treatment, as measured by the PFS and OS curves, with the area between both curves giving the PPS.

Efficacy data for teclistamab were obtained through matching-adjusted indirect comparisons (MAICs) to compare the efficacy of elranatamab with that of teclistamab, in order to adjust for the differences in key population characteristics between studies assessing the treatments [11,18,19,20]; these parameters included age, sex (for OS only), time since initial diagnosis, ISS stage, cytogenetic risk, extramedullary disease, number of prior lines of therapy, ECOG performance status, creatinine clearance, refractory status (penta-exposed; penta-refractory), and type of myeloma.

The relative effect of elranatamab compared to teclistamab, reported in the MagnetisMM-3 [18] and MajesTEC-1 [19] trials, respectively, in terms of time-to-event endpoints was quantified as a hazard ratio (HR) with a 95% CI. For this, the KM curves from the comparator trial were digitized following Guyot et al. (2012) [21]. For teclistamab, HR values of 0.60 and 0.55 were used for the OS and PFS curves, respectively.

TTD was included to capture treatment discontinuation due to reasons other than disease progression, such as adverse events, physician decision, or patient preference. TTD curves were fitted to characterize time on therapy within the PFS state, partitioning progression-free patients into on- and off-treatment cohorts.

For elranatamab, the median TTD was derived from MagnetisMM-3, while for teclistamab, the median TTD Kaplan–Meier curve was drawn from the MajesTEC-1 trial and digitized following Guyot et al. (2012) to obtain pseudo-individual-patient-level data, based on which the parametric fits were obtained [21].

For the base case, the treatment durations of teclistamab were estimated using an exponential distribution based on the reported median treatment duration. The median durations for TTD adopted in the base case analysis were 5.55 months for elranatamab and 8.50 months for teclistamab.

Adverse event (AE) data were obtained from published clinical trials of comparator treatments [11,18,19]. Given comparable follow-up durations, AE incidence rates at the longest available follow-up were used when reported for treatments [18,19]; otherwise, data obtained with shorter follow-ups were used [18,19].

**Figure 1:**
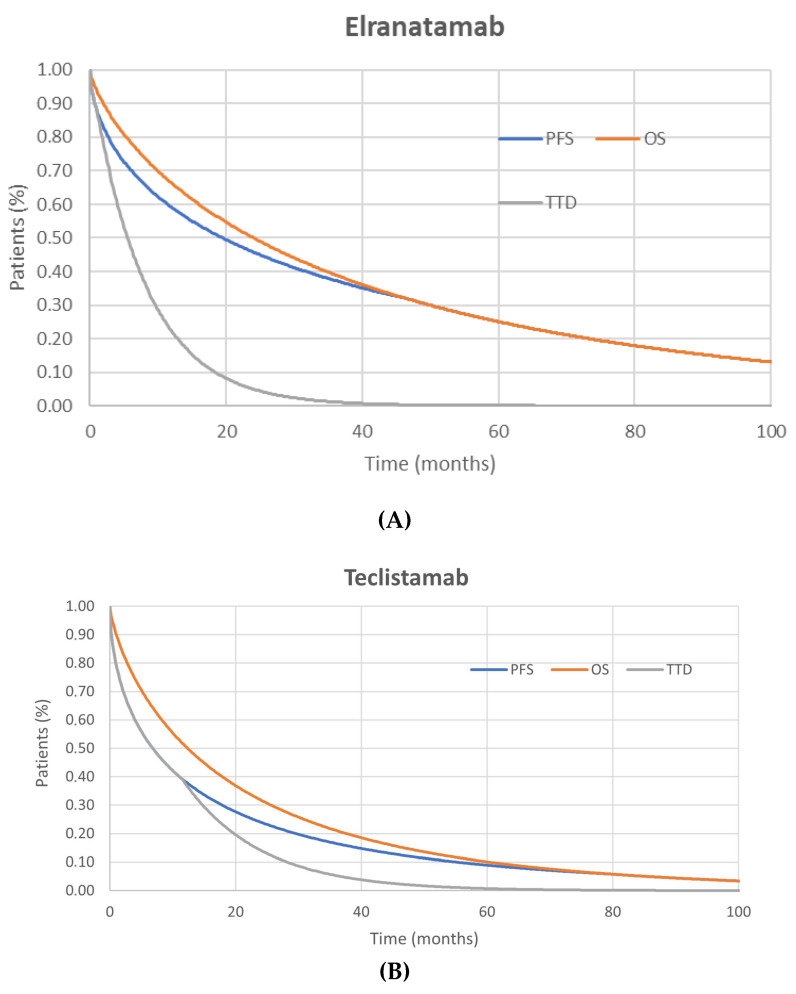
Progression-free survival (PFS), overall survival (OS), and time to discontinuation (TTD) Kaplan–Meier (KM) curves for elranatamab (**A**) and teclistamab (**B**).

The model included grade 3–4 AEs, except for adverse events of special interest (CRS and ICANS), for which all grades were considered due to their clinical and economic impact. In the base case, AEs with an incidence of ≥5% in either treatment arm were included, with higher thresholds tested in scenario analyses. Figure 1 presents the base case effectiveness model inputs for elranatamab and teclistamab, showing the mean estimated values (in years) for OS, PFS, and TTD.

Additional data on adverse events are reported in the following section on resource use and cost.

#### 2.1.2. Quality of Life

Health utility values were derived from patient-reported outcomes collected in the MagnetisMM-3 trial (median follow-up: 28.4 months) [18], based on the EQ-5D-5L questionnaire mapped to EQ-5D-3L using the algorithm of Hernández et al. (2023) [22]. Utilities of 0.71 for progression-free and 0.63 for post-progression states were applied to both treatments.

Disutilities and event durations for common AEs were estimated based on the MagnetisMM-3 data [11]. For AEs, an average disutility value of −0.028 was applied, which was estimated from the overall analysis.

### 2.2. Treatment Costs

The model considered the primary treatment cost, including pre-medication costs for primary treatments (where relevant), subsequent treatment costs, and administration costs.

#### 2.2.1. Primary Treatments

Ex-factory prices were obtained from the Gazzetta Ufficiale: Determina n. 75/2024 (GU Serie Generale n. 131, 6 June 2024) [23] for elranatamab and Determina n. 606/2023 (GU Serie Generale n. 238, 11 October 2023) [24] for teclistamab. The cost per treatment cycle was calculated based on the dosing schedules reported in the respective Summaries of Product Characteristics (SmPC) [25,26], and are reported in Tables S1 and S2.

Elranatamab is administered as two subcutaneous step-up doses (12 mg on Day 1 and 32 mg on Day 4), followed by 76 mg once weekly (QW) from Week 2 to Week 24, with a subsequent extension to every two weeks (Q2W) in responding patients after 24 weeks [25]. Teclistamab is given subcutaneously at 1.5 mg/kg QW, preceded by step-up doses of 0.06 mg/kg and 0.3 mg/kg, with the option to reduce dosing frequency to Q2W in patients achieving a sustained complete response for at least six months [26].

In the model, a QW-to-Q2W dosing switch was implemented for both treatments: all elranatamab-treated patients were assumed to transition to Q2W after 24 weeks [11] while, for teclistamab, the switch was assumed to occur in 46.1% of patients after 11.3 months [19,20,25], in line with published evidence.

#### 2.2.2. Subsequent Treatments

The model assumed that 65.8% of patients progressing to the post-progression state received subsequent treatment, accounted for cost purposes only, in accordance with data from the MagnetisMM-3 trial [11].

The composition, frequency, and costs [27] were defined and validated by two hematologists based on their clinical practice experience (Tables S3 and S4).

### 2.3. Administration Costs

Administration costs were retrieved from Italian NHS reference tariffs [28], as summarized in Table S5.

### 2.4. Healthcare Resource Use and Costs

The model included healthcare resource use derived from disease management across the PFS, PPS, and end-of-life health states, assuming utilization by all patients. Resource use in the PFS and PPS states included laboratory monitoring tests (complete blood count and biochemical tests performed according to routine and experts’ opinion), medical resource use (monthly outpatient physician visits), and hospitalization associated with treatment administration. Unit costs were sourced from the Tariffario Nazionale delle Prestazioni Ambulatoriali [28] and the Diagnosis-Related Group (DRG) reimbursement system [29], as reported in Table S6.

### 2.5. Adverse Events

The AE frequencies [11,18,19] and unit costs applied in the model are presented in Table S7, as well as AEs of special interest. AE-related costs were defined based on expert opinion and clinical practice, considering event-specific management pathways and resource use. Unit cost estimates were derived from the Tariffario Nazionale delle Prestazioni Ambulatoriali [28] and DRG tariffs [29].

A one-off end-of-life cost of EUR 2000 was included, based on the Italian literature [19], representing the average weekly cost of care for oncology patients not receiving palliative care during the last week of life [30].

Through a series of online meetings, all data inputs and model assumptions were validated by two hematologists, both of whom are recognized experts in RRMM at Italian university hospitals.

### 2.6. Sensitivity Analyses

In accordance with NICE methodological guidance for economic evaluations [31], deterministic and probabilistic sensitivity analyses were conducted to assess parameter uncertainty and the robustness of the results. In the deterministic sensitivity analysis (DSA), key parameters were varied individually by ±20% from their base case values to identify the main drivers of model outcomes. The probabilistic sensitivity analysis (PSA) captured joint parameter uncertainty through 5000 Monte Carlo simulations, with parameters sampled from pre-defined probability distributions and applied within the model framework to generate variability in outcomes.

## 3. Results

The results for the base case are shown in Table 1. In this analysis, elranatamab was associated with a total cost per patient of EUR 153,337, lower than the EUR 224,610 estimated for teclistamab, resulting in lifetime cost savings of EUR 71,273 per patient.

**Figure 2:**
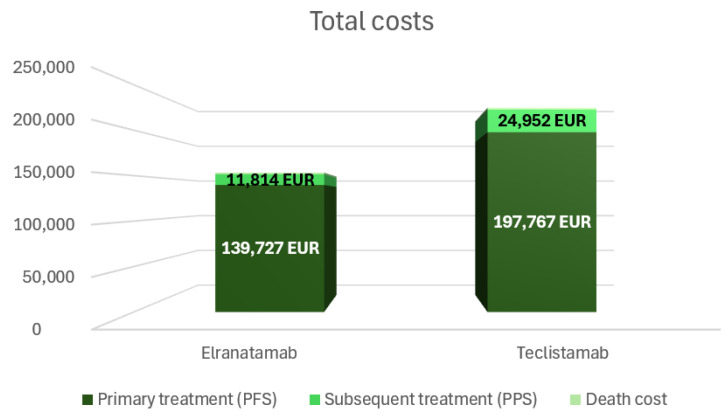
Distribution of costs by treatment phase (PFS, PPS, death cost) for elranatamab and teclistamab.

The primary cost driver for both treatments was the PFS phase, accounting for EUR 139,727 for elranatamab and EUR 197,767 for teclistamab, primarily due to drug acquisition costs (EUR 129,805 and EUR 187,744, respectively). PPS costs were also lower for elranatamab (EUR 11,814) compared with teclistamab (EUR 24,952), as were end-of-life care costs (EUR 1796 and EUR 1892, respectively); see Figure 2.

Regarding health outcomes, elranatamab provided longer survival and better quality-adjusted life expectancy, providing 3.31 life-years (LYs) and 2.33 QALYs per patient, compared with 1.83 LYs and 1.27 QALYs for teclistamab.

When comparing incremental effectiveness and costs, elranatamab generated 1.48 additional LYs and 1.06 additional QALYs relative to teclistamab, while resulting in cost savings of EUR 71,273 per patient over a lifetime horizon. Consequently, elranatamab was identified as a dominant therapeutic strategy, being both less costly and more effective than teclistamab in the management of patients with TCE/RRMM.

### 3.1. Sensitivity Analyses Results

#### 3.1.1. Probabilistic Sensitivity Analysis (PSA)

**Figure 3:**
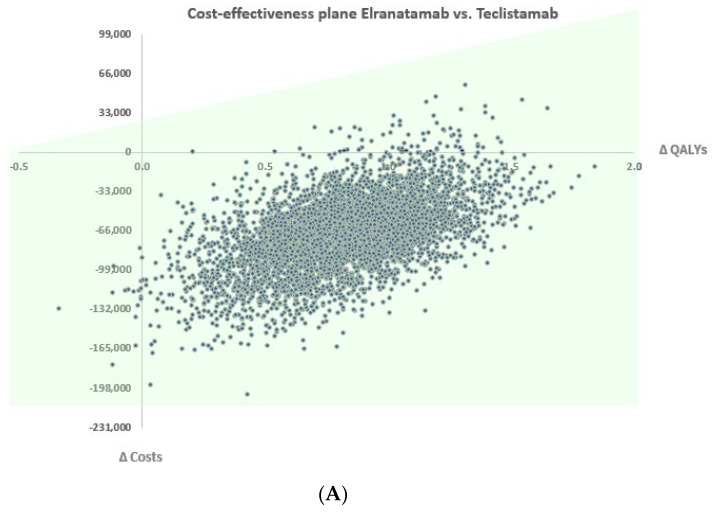

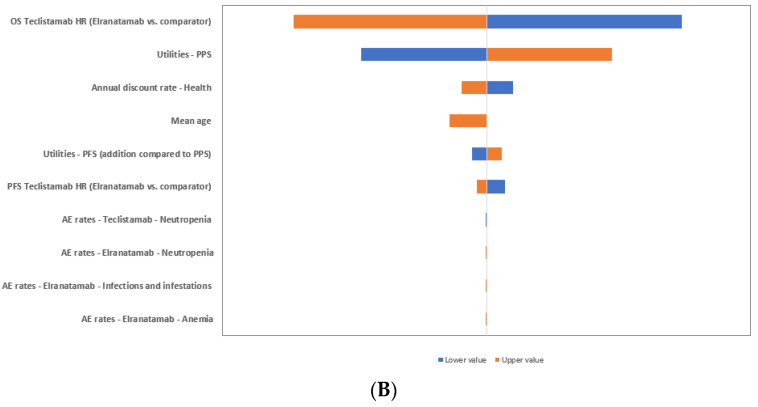
PSA and DSA results. The PSA (**A**) reveals the distribution of incremental costs and QALYs, with a 30,000 EUR/QALY WTP threshold highlighting the cost-effective area (in green). The DSA (**B**) identifies the HR for OS of teclistamab versus the comparator as the parameter with the largest impact on incremental QALYs, followed by PFS utilities, discount rate, and age. Overall, the results remain robust across plausible parameter variations. OS = overall survival, HR = hazard ratio, PFS = progression-free survival, PPS = post-progression survival, QALYs = Quality-Adjusted Life-Years, AE = adverse event.

PSA based on 5000 Monte Carlo iterations confirmed the robustness of the base case results (Figure 3A). Regarding the cost-effectiveness plan, nearly all simulated ICERs were in the south-east quadrant, indicating that elranatamab was consistently less costly and more effective than teclistamab. Across all iterations, elranatamab remained dominant, yielding mean incremental gains of approximately 0.85 QALYs and EUR 74,000 in cost savings per patient.

These findings confirm the high degree of certainty associated with the health and economic advantages of elranatamab in the treatment of TCE/RRMM.

#### 3.1.2. Deterministic Sensitivity Analysis (DSA)

The DSA results indicated that the OS hazard ratio for teclistamab versus the comparator was the parameter exerting the greatest influence on incremental QALYs, producing substantial variation in QALY outcomes when varied across its plausible ranges (Figure 3B).

Utilities during PFS also showed a non-negligible impact on the results. Health annual discount rate, the mean age, PFS utilities (addition compared PPS), and the PFS teclistamab HR had more modest impacts on incremental QALY. On the contrary, adverse event rates (including neutropenia, infections, and anemia for both treatments) did not have significant impacts on the results. Overall, despite parameter uncertainty, the variations observed across the analyzed inputs did not materially change the ranking of the scenarios, indicating that the model’s QALY outcomes remain robust to reasonable fluctuations in clinical and utility assumptions.

## 4. Discussion

Current regimens for TCE/RRMM are associated with suboptimal outcomes and substantial healthcare resource utilization, highlighting the need for effective late-line therapies that can improve survival while limiting the associated economic burden.

In this context, several novel therapeutic alternatives—such as bispecific antibodies and CAR T-cell therapies—have emerged in recent years and demonstrated efficacy in heavily pre-treated patients. However, the present analysis did not include a cost-effectiveness assessment versus CAR T-cell therapies due to the absence of published MAICs at the time of model development.

Our cost-effectiveness analysis focuses on the comparison between elranatamab and teclistamab, evaluating their relative clinical benefits and economic impacts from the perspective of the Italian NHS. Specifically, elranatamab was found to be dominant with respect to teclistamab, delivering additional QALYs alongside lower lifetime costs from the Italian NHS perspective. The main findings show clear health outcomes gains with elranatamab compared to teclistamab (+1.48 QALYs; +1.06 LYs) and lower total costs (EUR −71,273 per patient), driven primarily by lower expenditures in the progression-free phase (notably drug acquisition) and, secondarily, by reduced post-progression and end-of-life costs.

Sensitivity analyses confirmed the robustness of these findings. All probabilistic simulations resulted in positive incremental QALYs, and deterministic sensitivity analyses consistently identified elranatamab as dominant across all tested scenarios. The cost-effectiveness plane showed that nearly all simulations fell within the south-east quadrant, indicating its greater effectiveness at lower cost.

Comparability between treatments was enhanced by the MAIC methodology. Moreover, recently published longer-term data for elranatamab and teclistamab suggest more favorable PFS and OS outcomes for elranatamab than those assumed in the base case, indicating that the present analysis may be conservative with respect to elranatamab’s relative effectiveness.

In the partitioned survival model applied in our analysis, patients may remain in the progression-free state while off treatment, and the clinical benefit (PFS/OS) is not assumed to end at the time of treatment discontinuation. In our study, elranatamab showed a shorter median TTD than teclistamab (5.55 months vs. 8.50 months); however, this does not imply an earlier loss of response, nor does it indicate that patients stopped responding upon treatment discontinuation.

This interpretation is consistent with findings presented at the most recent ASH Annual Meeting in a subgroup analysis from MagnetisMM-3 [32]. This post hoc analysis evaluated patients who experienced a prolonged interruption of elranatamab or permanently discontinued treatment while maintaining their response for more than 6 months. The authors reported that the majority of patients maintained their clinical response despite prolonged treatment interruption, demonstrating the durability of elranatamab responses. Furthermore, these data support the feasibility of interruptions to manage adverse events and allow treatment breaks without compromising the efficacy of elranatamab.

In our analysis, we incorporated the transition from QW to Q2W administration for both treatments, consistent with the evidence available at the time of model development. However, it is important to note that, according to the Italian SmPC20, patients receiving elranatamab who have completed at least 24 weeks of treatment on the Q2W schedule and have maintained their clinical response are eligible to further extend the dosing interval to once every four weeks (Q4W). This additional reduction in dosing frequency would be expected to further decrease treatment costs for elranatamab. Therefore, the cost estimates applied in our base case may be conservative, potentially underestimating the cost-effectiveness advantage of elranatamab relative to teclistamab. The findings of our study align with recent evidence highlighting the substantial clinical and economic value of anti-BCMA bispecific antibodies in the management of advanced-stage RRMM.

To our knowledge, this is the first published cost-effectiveness analysis of elranatamab versus teclistamab incorporating long-term data from the MagnetisMM-3 and MajesTEC-1 trials for comparator treatments.

A Spanish cost-effectiveness analysis [33] reporting the dominance of elranatamab over teclistamab is consistent with our conclusions; however, that study was based on clinical data with a shorter median follow-up for elranatamab (14.7 months). In contrast, our analysis incorporates longer-term clinical evidence, strengthening the existing findings and providing updated insights regarding the sustained value of elranatamab over time within the Italian healthcare context, thereby supporting decision-making for the Italian NHS.

Compared with other MAICs [34], our work is more methodologically robust as it is not limited to cost per responder but incorporates all clinically relevant outcomes from pivotal trials. While responder-based analyses offer informative snapshots, they capture only part of the treatment effect. By accounting for the outcomes of patients who do not fully respond, our evaluation more closely reflects real-world clinical practice.

In addition, the integration of multiple clinical parameters enabled the derivation of key comparative metrics, such as LYs and QALYs. The availability and use of long-term cost-effectiveness data represents a notable strength relative to studies adopting alternative designs, providing more robust support for HTA and reimbursement decision-making.

We acknowledge that international real-world evidence, including data from the Intergroupe Francophone du Myélome (IFM) compassionate use program, provides an important perspective on the effectiveness of elranatamab in routine clinical practice. The IFM cohort reflects outcomes observed in a population that is generally more heavily pre-treated and clinically more complex than that characterized by the patients enrolled in the pivotal MagnetisMM-3 trial, with a higher burden of adverse prognostic factors. In this context, less favorable effectiveness outcomes are clinically plausible and should be interpreted according to the underlying baseline risk profile, rather than as a direct contradiction of trial results [35].

Our analysis did not aim to extrapolate effectiveness across all possible real-world settings but, rather, to estimate the relative cost-effectiveness of elranatamab versus teclistamab using the most robust comparative clinical evidence available at the time of model development.

Moreover, additional real-world studies provide complementary evidence supporting both the clinical and economic value of elranatamab. Data from the ALTITUDE-1 study indicate that initiation of elranatamab is not associated with an increase in overall healthcare expenditures, reporting stable monthly per-patient costs [36]. Further analyses have reported lower total costs and lower cost per month of progression-free survival compared with teclistamab [37], as well as a limited three-year budget impact and favorable economic value relative to other RRMM therapies [38].

The totality of the available evidence, both clinical and economic, remains consistent with the positioning of elranatamab as a clinically meaningful and economically efficient option in TCE/RRMM. Our study has several limitations, including those inherent to long-term cost-effectiveness modeling, such as survival curve extrapolation and the PSM’s three-state structure, which simplifies disease pathways and may not capture all clinical nuances.

Limitations also arise from the lack of head-to-head clinical comparisons, necessitating indirect comparisons and unanchored MAICs, which can only adjust for mutually reported baseline characteristics.

We also recognize limitations in estimating healthcare resource use and costs, as real-world data for elranatamab and teclistamab in Italy are lacking and, thus, expert opinion and protocol-driven assumptions were required for some inputs. While these estimates had a minimal impact on the overall results, certain differences may arise in clinical practice.

Finally, uncertainty in estimating subsequent treatments and costs is a common challenge in later lines of therapy with no clear recommendations. In this regard, further indirect evidence is needed to assess the impacts of these alternatives on costs and health outcomes.

## 5. Conclusions

The presented cost-effectiveness analysis revealed that elranatamab represents a financially favorable option for adult patients with TCE/RRMM in Italy when compared to teclistamab, delivering improved clinical outcomes and showing potential to reduce overall treatment costs.

## Figures and Tables

**Table 1:** Total costs, clinical outcomes, and incremental results for elranatamab and teclistamab in the treatment of patients with TCE/RRMM.

Parameters	Treatment Regimen	
	Elranatamab	Teclistamab
Cost and health outcomes		
Total costs	EUR 153,337	EUR 224,610
Progression-free survival	EUR 139,727	EUR 197,767
Post-progression survival	EUR 11,814	EUR 24,952
Death	EUR 1796	EUR 1892
LYs	3.31	1.83
QALYs	2.33	1.27
Incremental results		
Incremental costs	Reference	EUR −71,273
Incremental LYs	Reference	1.48
Incremental QALYs	Reference	1.06
ICER (EUR/LY)	Reference	Dominant
ICER (EUR/QALY)	Reference	Dominant

(LYs = life-years, QALYs = Quality-Adjusted Life-Years, ICER = Incremental Cost-Effectiveness Ratio).

## Data Availability

The original contributions presented in this study are included in the article/Supplementary Materials. Further inquiries can be directed to the corresponding author.

## Supplementary Materials

The following supporting information can be downloaded at: https://www.mdpi.com/article/10.3390/cancers18071070/s1, Figure S1: Model structure including three health states: progression-free survival (PFS), progressed disease, and death; Table S1: Primary treatment unit costs; Table S2: Subsequent treatment frequencies; Table S3: Subsequent treatment unit costs; Table S4: Administration unit costs; Table S5: Healthcare resource use and unit costs applied in the model; Table S6: Adverse events—incidence and unit costs; Table S7: AEs of special interest—incidence and unit costs.

## Abbreviations

The following abbreviations are used in this manuscript:

MM	Multiple myeloma
TCE/RRMM	Triple-class exposed relapsed/refractory MM
NHS	Italian National Health Service
RRMM	Relapsed/refractory multiple myeloma
PSM	Partitioned survival model
PFS	Progression-free survival
PPS	Post-progression survival
OS	Overall survival
TTD	Time to treatment discontinuation
ICER	Incremental Cost-Effectiveness Ratio
CHEERS	Consolidated Health Economic Evaluation Reporting Standards
AIC	Akaike Information Criterion
BIC	Bayesian Information Criterion
MAICs	Matching-adjusted indirect comparisons
HR	Hazard ratio
AE	Adverse event
SmPC	Summaries of Product Characteristics
QW	Once weekly
Q2W	Once every two weeks
DRG	Diagnosis-Related Group
DSA	Deterministic sensitivity analysis
PSA	Probabilistic sensitivity analysis
LYs	Life-years
QALYs	Quality-Adjusted Life-Years
Q4W	Once every four weeks
