# Supplementary material for: Cost-Effectiveness of Elranatamab Versus Teclistamab for the Management of Patients with Triple-Class Exposed Relapsed/Refractory Multiple Myeloma in Italy
Source: Cancers (Basel). 2026 Mar 25;18(7):1070. doi: 10.3390/cancers18071070 (PMC13071960; doi:10.3390/cancers18071070)

# Cost-Effectiveness of Elranatamab Versus Teclistamab for the Management of Patients with Triple-Class Exposed Relapsed/Refractory Multiple Myeloma in Italy

## CEA in RRMM: elranatamab vs teclistamab

Cirino Botta[1], Giorgio Lorenzo Colombo[2,3], Sergio Di Matteo[4], Chiara Martinotti[4], Emma Lucia Fogliati[4], Giacomo Matteo Bruno[2], Giuseppe Novelli[5], Roberto Di Virgilio[5], Barbara Veggia[5], Sara Galimberti[6]

1. Department of Health Promotion, Mother and Child Care, Internal Medicine and Medical Specialties, University of Palermo, Palermo, Italy
2. Department of Drug Sciences, University of Pavia, Italy
3. CEFAT - Center of Pharmaceuticals Economics and Medical Technologies Evaluation, Department of Drug Sciences University of Pavia, Italy
4. Center of Research, SAVE Studi - Health Economics and Outcomes Research, Milan, Italy
5. Pfizer Italia s.r.l., Rome, Italy
6. Department of Clinical and Experimental Medicine, section of Hematology, University of Pisa, Italy

Giorgio Lorenzo Colombo: giorgio.colombo@unipv.it
Chiara Martinotti: chiara.martinotti@savestudi.it
Emma Lucia Fogliati: emma.fogliati@savestudi.it
Giacomo Matteo Bruno: giacomomatteo.bruno@unipv.it
Giuseppe Novelli: giuseppe.novelli@pfizer.com
Sara Galimberti: sara.galimberti@unipi.it

**Table S1.** Primary treatment unit costs

| Primary treatment | Cost per treatment cycle |
|---|---|
| Elranatamab (step-up) | EUR 2.913,00 |
| Elranatamab | EUR 5.032,00 |
| Teclistamab (step-up) | EUR 816,74 |
| Teclistamab | EUR 4.165,38 |

**Table S2.** Subsequent treatment for elranatamab and teclistamab – frequency

| Subsequent treatment | Frequency |
|---|---|
| Melflufen | 10% |
| Bortezomib, cisplatin, cyclophosphamide dexamethasone, etoposide | 10% |
| Carfilzomib, dexamethasone | 5% |
| Bortezomib, dexamethasone | 5% |
| Selinexor, dexamethasone | 10% |
| Elotuzumab, pomalidomide, dexamethasone | 20% |
| Talquetamab | 40% |

**Table S3.** Subsequent treatment unit cost for elranatamab and teclistamab

| Subsequent treatment | Cost unit[28] |
|---|---|
| Melflufen | EUR 10.53 |
| Bortezomib | EUR 553.10 |
| Cisplatin | EUR 23.23 |
| Cyclophosphamide | EUR 8.40 |
| Dexamethasone | EUR 6.14 |
| Etoposide | EUR 7.10 |
| Carfilzomib | EUR 1,147.17 |
| Selinexor | EUR 8,057.98 |
| Elotuzumab | EUR 1,496.50 |
| Pomalidomide | EUR 5,926.38 |
| Talquetamab (step-up) | EUR 343.64 |
| Talquetamab | EUR 4,581.92 |

**Table S4**. Administration unit cost

| Administration mode | Unit cost |
|---|---|
| Oral | EUR 0 |
| Injection | EUR 85 |
| Intravenous injection | EUR 150 |

**Table S5.** Healthcare resource use and unit costs applied in the model

| Healtcare resource | Unit cost |
|---|---|
| *Assumed for all treatments* | |
| Complete blood count | EUR 3.17 |
| Biochemical test | EUR 2.17 |
| Physicians' office visit | EUR 20.66 |

| | |
|---|---|
| *Treatment-specific* | |
| Hospitalization (outpatient day) | EUR 371.00 |

**Table S6.** Adverse events: incidence and unit cost

| Adverse events | Elranatamab | Teclistamab | Cost |
|---|---|---|---|
| Acute kidney injury | 0.0% | 5.0% | EUR 7,185.00 |
| Alanine aminotransferase increased | 0.0% | 2.5% | EUR 1,238.00 |
| Anemia | 37.4% | 35.0% | EUR 1,212.00 |
| Aspartate aminotransferase increased | 0.0% | 2.5% | EUR 1,238.00 |
| Asthenia | 0.0% | 5.0% | EUR 16.00 |
| Bacteremia | 1.6% | 0.0% | EUR 5,493.00 |
| Blood alkaline phosphatase increased | 0.0% | 1.8% | EUR 1,171.00 |
| Bone pain | 0.0% | 2.5% | EUR 252.00 |
| COVID-19 | 15.4% | 12.1% | EUR 4,481.00 |
| Decreased appetite | 8.0% | 0.6% | EUR 8.00 |
| Diarrhea | 1.6% | 3.6% | EUR 959.00 |
| Dyspnea | 0.0% | 5.0% | EUR 6,764.00 |
| Fatigue | 3.3% | 2.4% | EUR 16.00 |
| Hypercalcemia | 0.0% | 2.5% | EUR 1,653.00 |
| Hypokalemia | 10.6% | 4.8% | EUR 1,653.00 |
| Hypertension | 0.0% | 5.5% | EUR 936.00 |
| Hyponatremia | 0.0% | 7.5% | EUR 1,653.00 |
| Infections and infestations | 42.3% | 32.5% | EUR 5,493.00 |
| Neurotoxicity | 0.0% | 0.0% | EUR 1,272.00 |
| Neutropenia | 49.6% | 65.0% | EUR 524.00 |
| Pneumonia | 8.1% | 12.7% | EUR 2,016.00 |
| Pyrexia | 4.1% | 0.6% | EUR 2,086.00 |
| Sepsis | 6.5% | 0.0% | EUR 5,493.00 |
| Sinusitis | 1.6% | 0.0% | EUR 1,703.00 |

| | | | |
|---|---|---|---|
| Thrombocytopenia | 23.6% | 30.0% | EUR 51.00 |
| Urinary tract infection | 3.3% | 0.0% | EUR 1,657.00 |

**Table S7.** AEs of special interest: incidence and unit cost

| Other AEs of special interest | Elranatamab | Teclistamab | Cost |
|---|---|---|---|
| Neurotoxicity (grade 1-2) | 3.4% | 5.5% | EUR 2,077.00 |
| CRS (grade 1-2) | 57.7% | 65.0% | EUR 1,836.00 |

**Figure S1.** Model structure including three health states: progression-free survival (PFS), progressed disease, and death

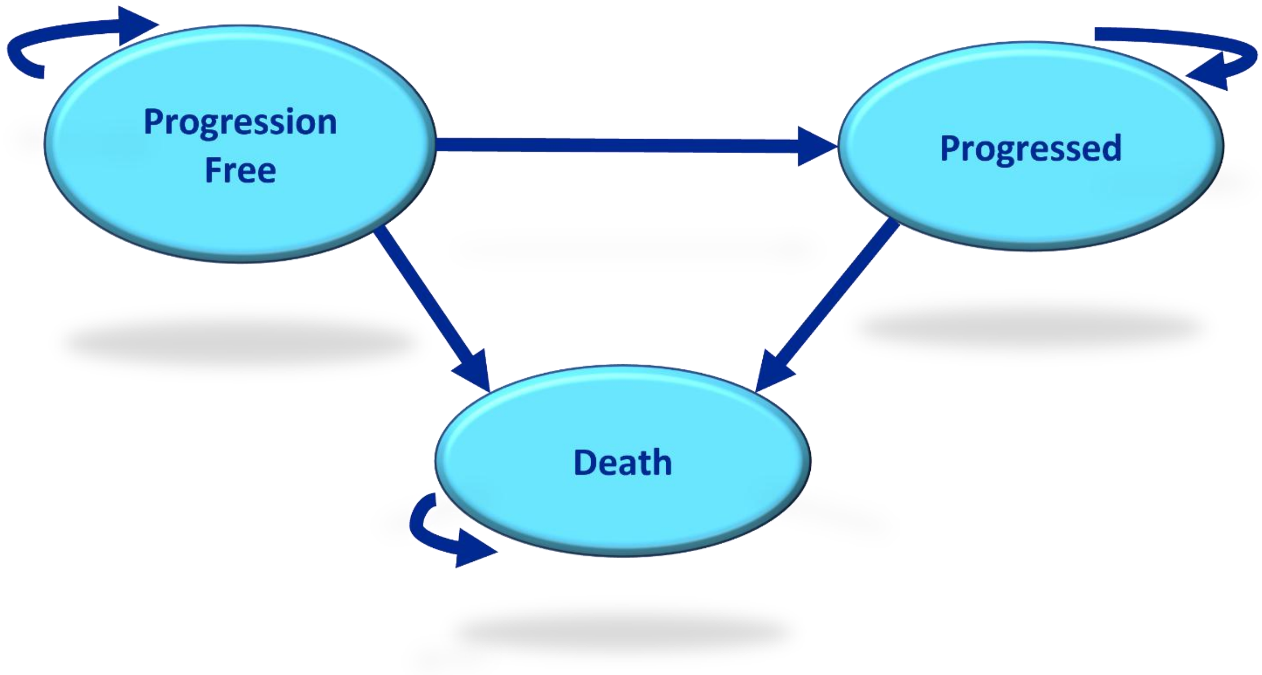

Supplement: Supplementary file 1 [file cancers-18-01070-s001.zip › cancers-4155248-supplementary.pdf]
